# SARS-CoV-2 does not have a strong effect on the nasopharyngeal microbial composition

**DOI:** 10.1038/s41598-021-88536-6

**Published:** 2021-04-26

**Authors:** Tzipi Braun, Shiraz Halevi, Rotem Hadar, Gilate Efroni, Efrat Glick Saar, Natahan Keller, Amnon Amir, Sharon Amit, Yael Haberman

**Affiliations:** 1 Pediatric Gastroenterology, Hepatology and Nutrition Unit, The Edmond and Lily Safra Children’s Hospital, Sheba Medical Center, Affiliated with the Tel-Aviv University, Tel Hashomer, Israel; 2 Clinical Microbiology, The Chaim Sheba Medical Centre, Ramat-Gan, Israel; 3 Sakler Faculty of Medicine, Tel Aviv University, Tel Aviv, Israel; 4 Cincinnati Children’s Hospital Medical Center and the University of Cincinnati College of Medicine, Cincinnati, OH USA

**Keywords:** SARS-CoV-2, Microbiome

## Abstract

The coronavirus disease 2019 (COVID-19) has rapidly spread around the world, impacting the lives of many individuals. Growing evidence suggests that the nasopharyngeal and respiratory tract microbiome are influenced by various health and disease conditions, including the presence and the severity of different viral disease. To evaluate the potential interactions between Severe Acute Respiratory Syndrome Corona 2 (SARS-CoV-2) and the nasopharyngeal microbiome. Microbial composition of nasopharyngeal swab samples submitted to the clinical microbiology lab for suspected SARS-CoV-2 infections was assessed using 16S amplicon sequencing. The study included a total of 55 nasopharyngeal samples from 33 subjects, with longitudinal sampling available for 12 out of the 33 subjects. 21 of the 33 subjects had at least one positive COVID-19 PCR results as determined by the clinical microbiology lab. Inter-personal variation was the strongest factor explaining > 75% of the microbial variation, irrespective of the SARS-CoV-2 status. No significant effect of SARS-CoV-2 on the nasopharyngeal microbial community was observed using multiple analysis methods. These results indicate that unlike some other viruses, for which an effect on the microbial composition was noted, SARS-CoV-2 does not have a strong effect on the nasopharynx microbial habitants.

## Introduction

Severe Acute Respiratory Syndrome Corona 2 (SARS-CoV-2)^1,2^ is the coronavirus responsible for the 2019–2020 pandemic. It infects the upper respiratory tract nasopharynx, and is transmitted mostly by tiny droplets and aerosols^2^, with some evidence suggesting also a fecal mode of transmission^3^. Due to low cross-protective immunity from related viral infections, SARS-CoV-2 transmissibility is high, hence facilitating widespread person-to-person transmission^4,5^. In fact, this SARS-CoV-2 has the highest transmissibility when compared to previous pandemics^6,7^. However, its clinical course is highly variable between subjects, and factors contributing to this heterogeneity are not yet entirely known.

Previous studies have shown that different environmental factors, such as smoking, affect the upper respiratory microbial composition^8^. Additionally, studies have shown a correlation between respiratory tract microbial composition and the severity of various viral diseases, including respiratory syncytial virus in children^9^ and influenza^10^. Others have observed reduced alpha diversity in the nasopharynx of patients during viral diseases^11^.

Two recent studies have attempted to identify differences between the nasopharyngeal microbiota of SARS-CoV-2 patients compared to healthy controls, with mixed results. One cohort, used 16S amplicon sequencing and included 40 patients, reported no significant differences between SARS-CoV-2 positive and negative subjects^4^. The second study used the direct Oxford Nanopore long-read third generation sequencing and included 50 patients suspected for COVID-19, and found some differences between those subjects that were positive and negative for SARS-CoV-2^12^. Here, we used 16S amplicon sequencing to define differences also by using longitudinal samples, which supplement the other two previous studies that used cross-sectional sampling.

## Results

### Cohort characteristics

**Figure 1:**
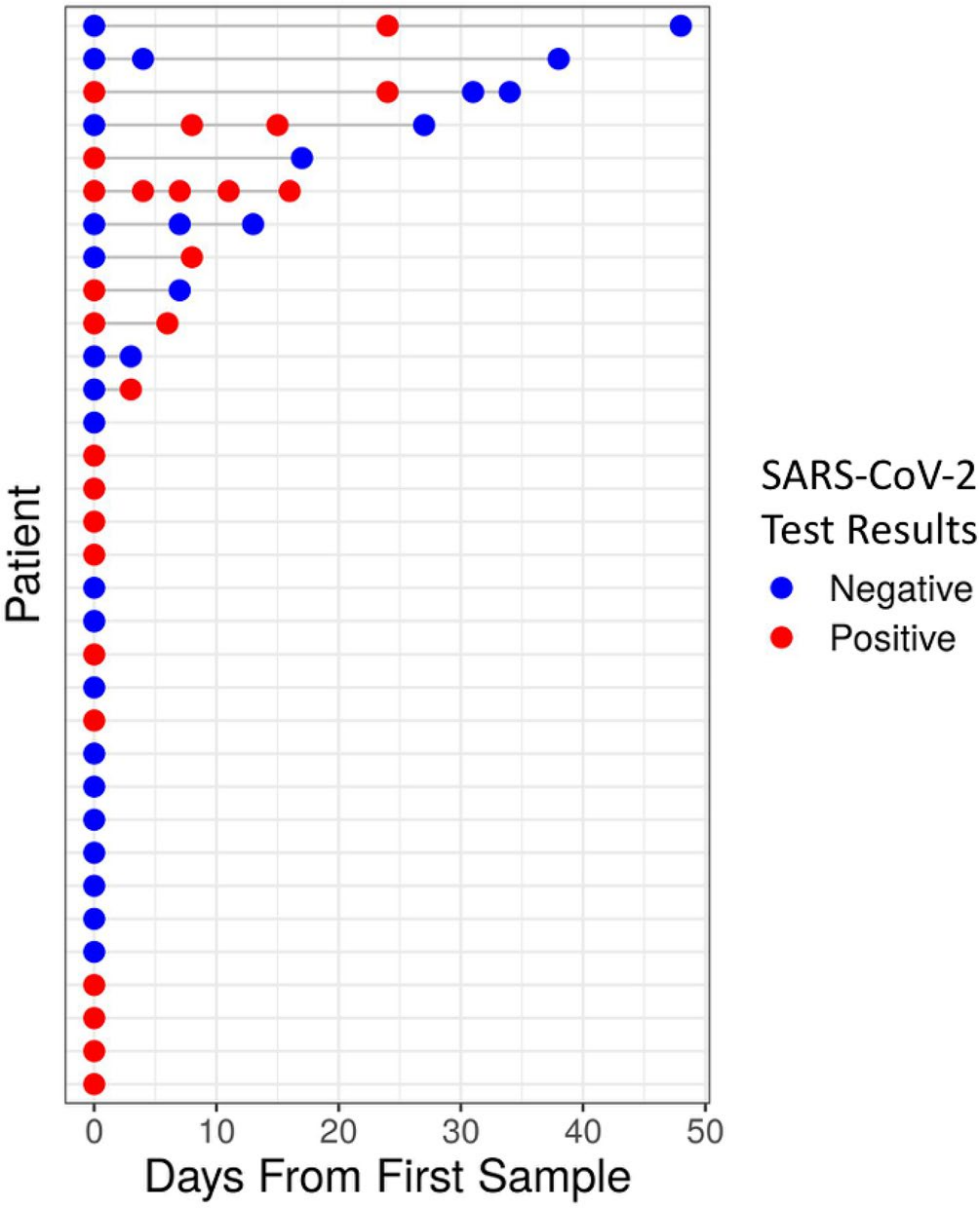
Longitudinal cohort of 55 samples from 33 subjects confirmed or suspected as being SARS-CoV-2 positive. Each row corresponds to an enrolled subject, over the number of days since first sample included in this study. Colors represent SARS-CoV-2 test results for a specific sample, with red and blue indicating SARS-Cov-2 positive and negative samples respectively.

55 Nasopharyngeal samples from 33 subjects, confirmed or suspect for COVID-19, were processed by 16S rRNA amplicon sequencing in parallel to SARS-CoV-2 testing by RT-PCR in the clinical microbiology lab. Twelve of the 33 patients had longitudinal samples with 2–5 samples each (Fig. 1). Overall, we had 29 SARS-CoV-2 negative samples and 26 SARS-CoV-2 positive samples, with 21 of the 33 patients having at least one positive SARS-CoV-2 sample. Patients had a median age of 52 years and 56% were male (Table 1).

**Table 1:** Cohort characteristics.

	Subjects (n = 33) with total 55 samples	
Age median (IQR)	n = 32	52 (30, 68)
Gender (male) %	n = 32	18 (56%)
SARS-CoV-2 positive ≥ 1 sample	n = 33	21 (64%)

### Factors effecting nasopharyngeal microbial composition

**Figure 2:**
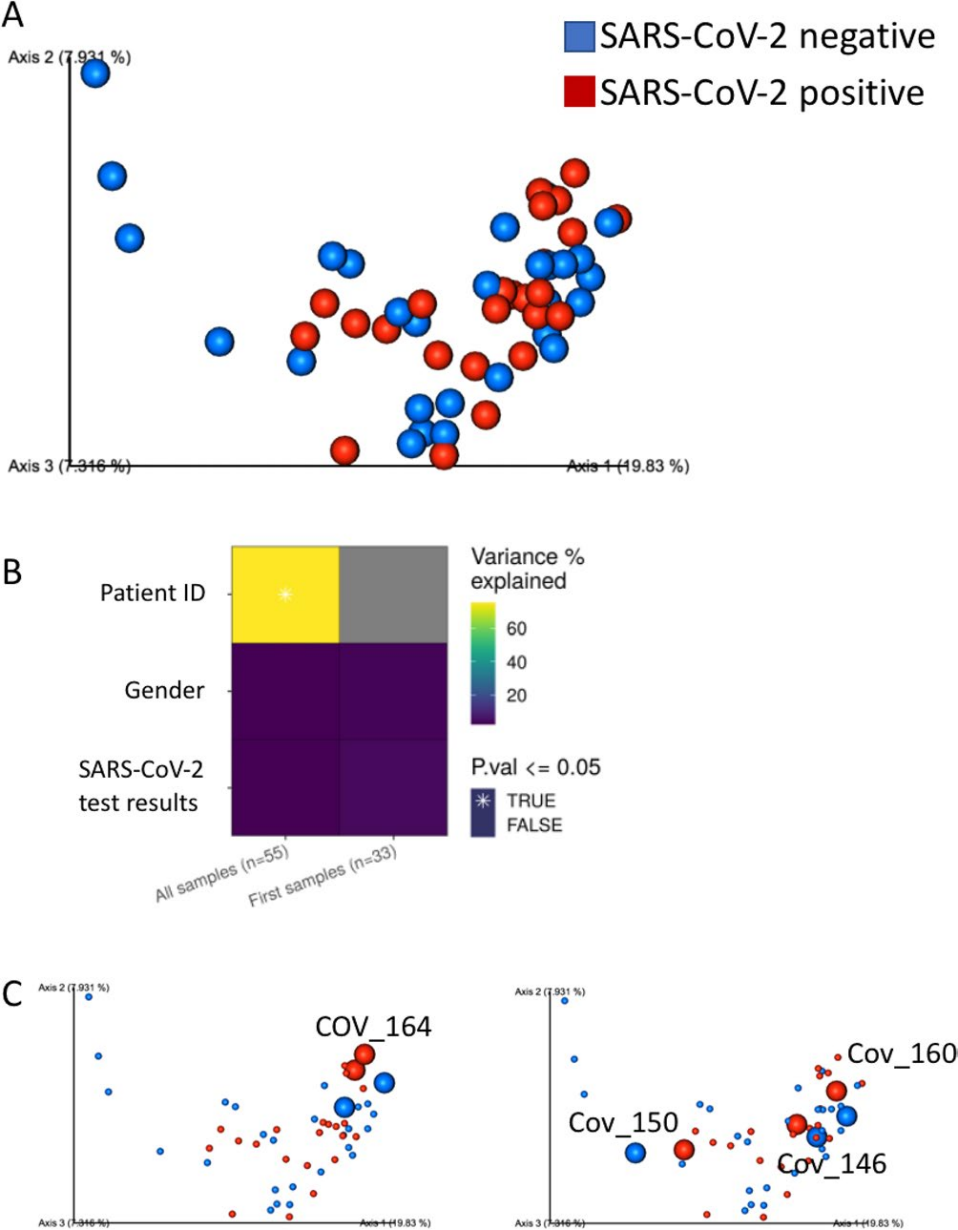
Personal variation has the strongest effect on nasopharyngeal microbial composition. (**A**) Unweighted UniFrac PCoA plot of all samples, colored by SARS-CoV-2 test results. (**B**) PERMANOVA analysis of microbial variance explained by subject (patient ID), gender and SARS-CoV-2 test result, using all longitudinal samples, as well as the first samples from each subject. * indicates statistical significance with *p* ≤ 0.05., and n is shown in brackets. (**C**) Unweighted UniFrac PCoA plot of all samples, colored by SARS-CoV-2 test results. SARS-CoV-2 positive and negative samples from specific subjects with longitude sampling are marked with increased size and labeled with the subject ID, showing clustering is driven by subject ID rather than SARS-CoV-2 test results.

Unweighted unifrac based PCoA was used to visually explore samples similarity and variations. As can be seen, SARS-CoV-2 testing result does not seem to have a strong effect, as samples do not cluster by COVID-19 test results (Fig. 2A). The contribution of the various factors to the microbial composition was quantified using a PERMANOVA test (see “Methods”), either using all of the samples, or by using only single sample per subject to avoid personal bias. Inter-personal variation, indicated by the patient ID, was highly important, explaining 76% of microbial variation (*p *value = 0.001), while SARS-CoV-2 test results, and gender did not have a significant effect, with a *p* value > 0.2 (Fig. 2B) using either all samples or only one sample per patient. The strong effect of patient identity on the microbial composition can be observed in the PCoA, where samples from the same patient clustered close to each other, regardless of COVID-19 test results (Fig. 2C).

### Inability to detect a strong contribution of COVID-19 results on microbial composition

**Figure 3:**
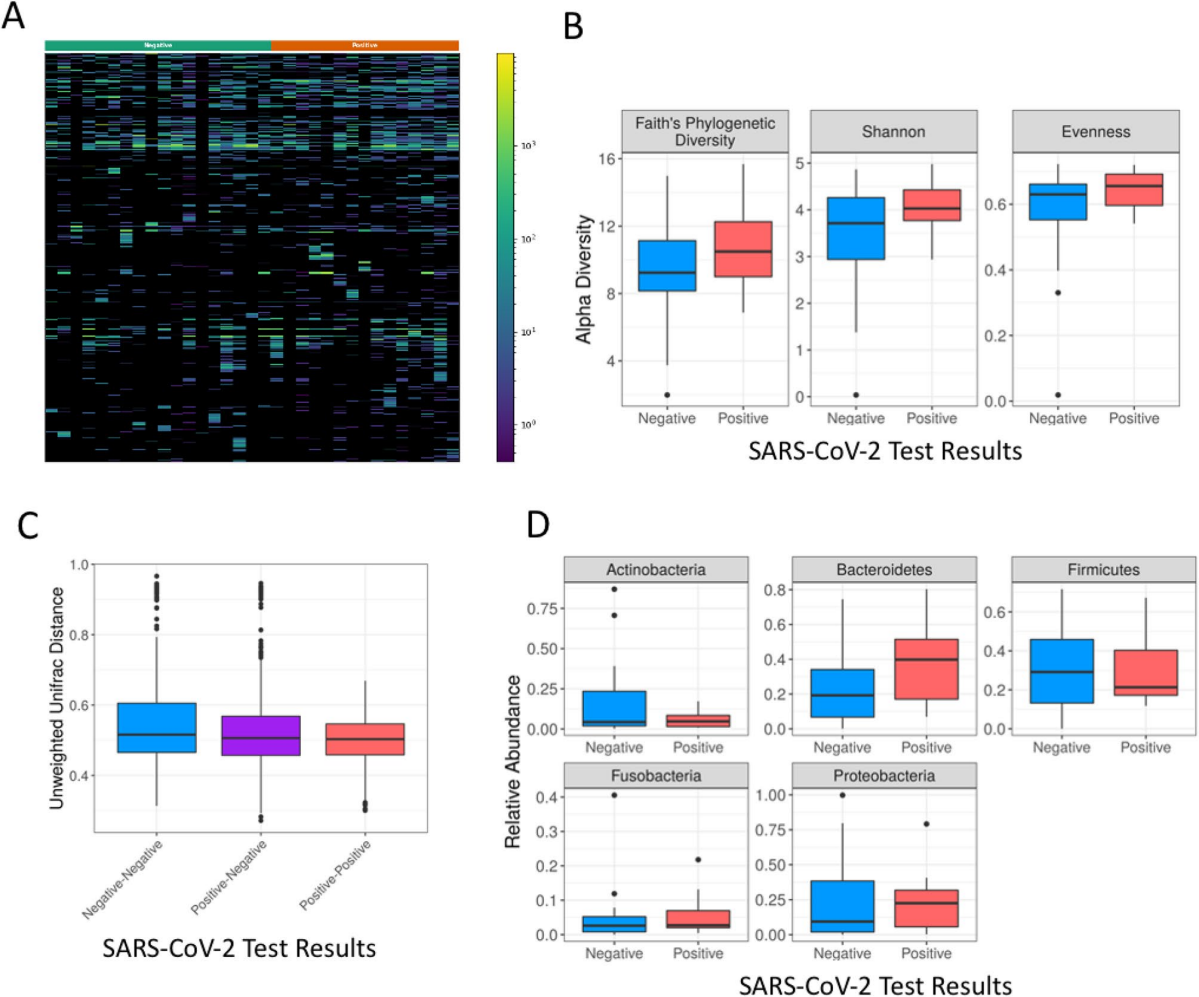
SARS-CoV-2 test result does not seem to have a strong effect on the nasopharyngeal microbial composition. (**A**) Heatmap showing all observed ASVs using the first sample from each subject. Each row represents a different ASV and each column a different sample. SARS-CoV-2 test results are noted in the color bar above (green for negative and orange for positive). (**B**) Boxplots of alpha diversity values, using the first sample from each subject, by SARS-CoV-2 test result. Three different alpha diversity measures were used—Faith’s phylogenetic diversity, Shannon, and evenness, as indicated. There were no significant differences in any of the measures tested (*p* value > 0.1). (**C**) Boxplot of unweighted unifrac distances between positive-positive, negative-negative and positive–negative sample pairs, using only the first sample per subject. No significant difference was detected, (PERMANOVA *p* value of 0.21). (**D**) Boxplots of the relative abundance of the five most abundant phyla by SARS-CoV-2 test result, using only the first sample per subject (Wilcoxon rank sum test *p* value > 0.05).

Since the microbial compositional analysis showed no difference between COVID-19 positive and negative samples, other methods were used to try and detect such differences. To avoid personal bias, we used only one sample per patient. A heatmap visualization showed no overt difference between the two groups (Fig. 3A). There was also no significant difference between the groups in any of the alpha diversity measures used including Faith's phylogenetic diversity, Shannon and evenness (Fig. 3B), with Wilcoxon rank sum test *p* values > 0.1 for all alpha diversity measures. An unweighted unifrac distance analysis also did not detect significant difference within or between positive and negative samples (Fig. 3C), with a PERMANOVA *p* value of 0.21. Similarly, we did not detect a significant difference in the relative abundance of the five most abundant phyla (Fig. 3D), (Wilcoxon rank sum test, *p* value > 0.06). We were also not able to detect any significant differences between specific amplicon sequence variants (ASVs) with an FDR of 0.25 using two different platforms—calour permutation rank mean test with dsFDR multiple hypothesis correction^13^, and maaslin2^14^ using all samples and controlling for patient ID as a random effect.

## Discussion

We were unable to detect a significant microbial pattern in the nasopharynx of SARS-CoV-2 positive subjects in comparison to SARS-CoV-2 negative subjects. This may imply that unlike what was seen with Rhinovirus infection, where a significantly higher diversity was observed in non-infected individuals compared to infected individuals^11^, COVID-19 does not have a strong effect on the nasopharyngeal microbial composition. In contrast, we have noticed a strong personal microbial signature, with samples from the same individual clustering together irrespective of whether samples were positive or negative for SARS-CoV-2, further implying that SARS-CoV-2 does not have strong effect on the nasopharynx microbiome. Two recent studies analyzed COVID-19 positive and negative nasopharyngeal samples with mixed results. The first used cross-sectional 16S amplicon sequencing^4^ and found no significant difference between infected and uninfected patient, similar to the results reported here. The second study also used a cross-sectional design and the direct Oxford Nanopore long-read third generation sequencing^12^. This study identified reduced microbial diversity and some differences in microbial communities. This significant difference was observed at the species level but not at the genus or family level. Therefore, the inability to detect differences in our current study may stem from the lower phylogenetic resolution of 16S rRNA amplicon sequencing compared to Oxford Nanopore derived long-reads, differences in populations, or sample sizes. It is also possible that the severity of certain viral diseases increases upon antibiotics administration, which reduce the microbial diversity, and potentially facilitate more rapid viral replication. To test for this possibility, larger studies that will take into account antibiotics administration during COVID-19 are required.

The longitudinal subset in our study included samples from different stages of the SARS-CoV-2 infection including pre- and post- SARS-CoV-2 positive results, corresponding to early and late infection stages, as well as negative tests obtained following SARS-CoV-2 infection. We did not observe any prominent effects of the infection during any of these stages. In contrast, we observed a strong personal effect, as was previously described in other studies of nasopharyngeal microbiome^4^. This result supports the validity of this cohort, showing that the lack of SARS-CoV-2 infection effect on the nasopharyngeal microbiome was not due to technical problems.

Our work has several strengths as it includes both cross sectional and longitudinal samples, and was based on samples obtained during routine screening for COVID19. Limitations include the limited cohort size, the use of 16S rRNA amplicon sequencing, and the limited clinical data. While there is a possibility that a cohort of patients with more severe cases will show a stronger effect, in such a cohort, it would be hard to assess how much of the difference is attributed to the presence of the virus itself or is due to the COVID-19 disease severity, treatment-related conditions, and medications. It is also possible that emerging new variants^15^ may alter the nasopharyngeal microbial composition, and more research is required to address such concern. Further research is needed to validate if and to what extent, COVID-19 infection influences the nasopharyngeal microbial composition, and disease outcome.

## Methods

### Study design and sample processing

Ethical approval for the study was granted by the Sheba Local Research Ethics Committee and all methods were performed in accordance with the relevant guidelines and regulations. Since this study used nasopharyngeal specimens already submitted to the microbiology core as part of clinical workup and without identifiable patient information other than age, gender, and viral results, an exemption from patient consent was granted from the Sheba Local Research Ethics Committee. The primary goal of this study was to characterize the microbial composition and diversity between individuals with positive and negative SARS-CoV-2 results, and to characterize the microbial dynamics within individuals for which we have longitudinal sampling.

We randomly included samples with no specific selection or exclusion criteria other than, when possible, obtaining several samples per subject as implicated (Fig. 1 and Table 1). Sterile swabs were used to collect the nasopharyngeal samples. Nasopharyngeal samples specimens were analyzed for SARS-CoV-2 presence at the Clinical Microbiology Lab at the Sheba Medical Center in Israel, between April and May 2020. Nasopharyngeal samples were collected into UTM (Copan) using sterile swabs and lysed with Universal LB lysis buffer (Seegene). Nucleic acids were extracted using StarMag universal cartridge kit (Seegene) on a Microlab Starlet (Hamilton) extraction robot, and RT-PCR of E, RdRp and N genes of the SARS CoV-2 virus was done using Allplex 2019 nCoV assay (Seegene) according to manufacturer's protocol. Nucleic acid extracts from samples were used in parallel also for broad-range high throughput 16S rRNA amplicon sequencing (16S-seq). Negative controls including swab blanks (sterile swabs), extraction blanks (reagents), and PCR controls were also included in the sampling and analyses. 55 unique specimens for 33 individuals passed processing, quality control, and filtering, and were included in this study.

### DNA extraction, PCR amplification, and sequencing

DNA extraction and PCR amplification of the variable region 4 (V4) of the 16S rRNA gene using Illumina adapted universal primers 515F/806R39 was conducted using the direct PCR protocol [Extract-N-Amp Plant PCR kit (Sigma-Aldrich, Inc.)] as previously described^16^. Briefly, PCRs were conducted in 96 wells plate [denaturation for 3 min at 94 °C; 35 cycles (98 °C, 60 s; 55 °C, 60 s; 72 °C, 60 s) followed by elongation for 10 min at 72 °C]. Positive amplicons were pooled in equimolar concentrations into a composite sample that was size selected (300–500 bp) using agarose gel to reduce non-specific products from host DNA. Sequencing was performed on the Illumina MiSeq platform with the addition of 20% PhiX, and generating paired-end reads of 175b in length in each direction.

### Microbiome data processing and analysis

Reads were processed in a data curation pipeline implemented in QIIME 2 version 2019.4^17^. Reads were demultiplexed according to sample specific barcodes. Quality control was performed by truncating reads after three consecutive Phred scores lower than 20. Reads with ambiguous base calls or shorter than 150 bp after quality truncation were discarded. Amplicon Sequence variant (ASV) detection was performed using Deblur^18^. Unweighted UniFrac was used as a measure of beta-diversity = between sample diversity^19^, using a phylogenetic tree generated by SEPP^20^. Faith's phylogenetic diversity, Shannon diversity and evenness were calculated in QIIME 2 as measures of alpha diversity. All samples were rarefied to 2000 reads for alpha and beta diversity analysis, to avoid sample size affect. The resulting distance matrix was used to perform a principal coordinate analysis (PCoA). heatmaps were generated using Calour version 2018.10.1 with default parameters^21^. Differentially expressed ASVs between positive and negative results were detected using one sample per subject, with a non-parametric rank mean test as implemented in Calour^21^ with dsFDR multiple hypothesis correction^13^ (FDR < 0.25). As a second approach to test for ASVs significantly associated with COVID-19 positive test, we used MaAsLin2 (Multivariate Association with Linear Models) R package version 1.0.0.^14^ with an FDR of 0.25. This approach used all samples (including multiple samples per subject when available), controlling for age, gender, and patient ID as random effects.

PERMANOVA: Quantifications of variance were calculated using PERMANOVA with the adonis function in the R package Vegan^22^, using 999 iterations, on the rarefied Unweighted UniFrac distance values. The total variance explained by each variable was calculated independently of other variables (that is, as the sole variable in the model).

### Data availability

The study datasets were deposited at the National Center for Biotechnology Information as BioProject PRJNA688646.
